# Study on freeze–thaw cyclic durability of reclaimed ceramic concrete in western high altitude region

**DOI:** 10.1038/s41598-026-42770-y

**Published:** 2026-03-10

**Authors:** Peng Kuan, Li Heyuqiu, Li Yaping

**Affiliations:** 1 School of Mechanical Engineering, Chengdu Technological University, Chengdu, 610031 People’s Republic of China; 2 Office of Cooperation and Development, Chengdu University of Technology, Chengdu, 610059 People’s Republic of China

**Keywords:** Ceramic aggregate recycled concrete, Freeze–thaw cycle, Wiener distribution probability, Life prediction, Engineering, Materials science

## Abstract

To achieve the resource utilization of waste ceramic particles and promote the application of recycled concrete in cold regions, this study systematically investigates the effects of waste ceramic particle replacement ratios on the freeze–thaw resistance and service reliability of recycled concrete. Six groups of specimens with ceramic particle replacement ratios of 0%, 20%, 40%, 60%, 80%, and 100% were designed. Freeze–thaw cycle tests were carried out using the rapid freezing method, with mass loss rate and relative dynamic elastic modulus as the key evaluation indicators to determine the optimal mix proportion. Scanning electron microscopy (SEM) was used to observe the evolution of the microstructure, and a reliability function was established based on the Wiener distribution probability model to predict the remaining service life of the optimally proportioned specimens. The results show that during freeze–thaw cycles, the relative dynamic elastic modulus of all specimens decreases continuously, while the mass loss rate exhibits a variation trend of initial increase followed by subsequent decrease. The specimens with a 20% ceramic particle replacement ratio demonstrate the best freeze–thaw resistance, failing only after 398 freeze–thaw cycles. Microstructural analysis reveals that at early freeze–thaw cycles, the damage is dominated by the deterioration of the surface interfacial transition zone (ITZ), whereas at high cycles, severe degradation occurs, including pore connectivity and the collapse of the cementitious network formed by hydration products. When the ceramic particle replacement ratio exceeds 20%, the freeze–thaw resistance of the specimens decreases significantly. This study reveals the freeze–thaw deterioration mechanism of recycled concrete incorporating waste ceramic particles as aggregates, and the findings provide a theoretical basis and technical support for the mix optimization and engineering application of such recycled concrete in cold regions.

## Introduction

With the rapid development of the global construction industry towards high-quality and large-scale directions, the global generation of construction and demolition (C&D) waste has shown a sharp upward trend, becoming a common environmental challenge faced by countries around the world. Such waste not only occupies substantial land resources but also causes soil, water, and air pollution through landfill leachate, dust diffusion, and other pathways, which stands in sharp contradiction to the concept of green, low-carbon, and sustainable development vigorously advocated by the international community. Against this backdrop, the recycling and reuse of waste concrete have emerged as a globally recognized solution—after standardized treatment, it can be reused as recycled aggregates in engineering construction. This approach not only significantly reduces reliance on natural aggregate extraction and raw material procurement costs, effectively alleviating the pressure of C&D waste disposal, but also realizes the material circulation of the concrete industry, providing crucial support for the sustainable development of the global construction sector^1^.

The global demand for ceramic tile production and consumption continues to rise, and the global issue of ceramic tile waste discharge has become increasingly prominent. According to statistics, the global annual output of ceramic tile waste has exceeded 120 million tons, with major tile-producing countries such as China, India, and Italy accounting for the majority of emissions^2^. Promoting the global resource utilization of such waste will bring significant economic and environmental benefits to engineering construction in various countries. Notably, the glaze layer on the surface of waste tiles endows them with unique performance advantages. The recycled ceramic concrete prepared from waste tiles exhibits excellent durability in terms of carbonation resistance and sulfate attack resistance^3–6^. Compared with ordinary concrete, its mechanical properties do not show obvious degradation, and the risk of alkali-aggregate reaction is much lower than that of traditional natural sand and gravel aggregates^7,8^, indicating broad application prospects in various global engineering scenarios.

At present, numerous scholars have conducted a series of studies on recycled concrete and recycled concrete with Waste ceramic particles aggregates. Siddique et al.^9^ used ceramic waste as fine aggregate to replace natural sand and found that when the replacement ratio ranges from 20 to 100%, the concrete strength is superior to that of the reference group, and the increase in porosity does not impair its freeze–thaw resistance^9^. Dobiszewska et al.^10^ confirmed through 150 freeze–thaw cycle tests that the addition of 10% (by mass of cement) ceramic coarse filler significantly improves the freeze–thaw resistance of concrete, and digital analysis of pore distribution can effectively evaluate its frost resistance^10^. Saber et al.^11^ showed that the comprehensive performance of concrete is optimal when 25% of natural coarse aggregate is replaced by ceramic tile waste. After 56 freeze–thaw cycles, the concrete still maintains good stability. Life cycle assessment indicates that its global warming potential is reduced by 25%, and the total cost over a 60-year service life can be decreased by 45%-50%^11^. Khalil et al.^12^ conducted tests with three replacement ratios (25%, 50%, and 75%) and found that replacing brick aggregate with ceramic tile waste can significantly improve the compressive strength and splitting tensile strength of concrete while enhancing water absorption performance, achieving both environmental and technical benefits^12^. Fathifazl et al.^13^ explored the influence of ceramic tile aggregate replacement ratio on the compressive, tensile, and flexural strengths of concrete. Single-factor analysis of variance (*p* < 0.05) verified that the replacement ratio has a significant effect on concrete performance, providing a quantitative reference for engineering applications^13^. In addition, other existing studies have revealed the working mechanism and failure law of concrete from different angles.^14–18^

In response to the existing research gaps in the durability assessment and service life prediction of recycled concrete incorporating Waste ceramic particles aggregates, the present study focuses on recycled concrete with Waste ceramic particles aggregates as the core research subject. Guided by the test standards and evaluation protocols for the freeze–thaw cycle performance of ordinary concrete (as specified in relevant engineering codes), this research prioritizes the systematic exploration of the evolutionary laws governing the freeze–thaw resistance of such recycled concrete under cyclic freeze–thaw conditions. Two key durability metrics—relative dynamic elastic modulus and mass loss rate—are selected as the core evaluation indicators, given their high sensitivity to freeze–thaw-induced damage in concrete materials. The Wiener distribution probability model, which is widely recognized for its superior adaptability in characterizing gradual performance degradation processes of engineering materials, is employed to construct a quantitative model describing the performance degradation behavior of specimens prepared with the optimal mix proportion. Furthermore, based on the established degradation model, a reliability function is developed to quantitatively elucidate the intrinsic correlation between the reliability level of the test specimens and the cumulative number of freeze–thaw cycles. This analytical framework not only enables the accurate characterization of the dynamic reliability evolution of the material during freeze–thaw exposure but also provides robust theoretical support and practical technical references for the precise prediction of the remaining service life of Waste ceramic particles aggregate recycled concrete in freeze–thaw-prone engineering environments.

## Raw materials and test scheme

### Raw materials

The raw materials used in this test are as follows (Table 1): (1) Cement: Qilianshan brand 42.5 ordinary Portland cement produced was used. (2) Recycled aggregates: Self-made recycled aggregates with good gradation were used (Tables 2, 3). (3) Natural coarse aggregate: Natural coarse aggregate is crushed stone provided by a commercial concrete company in China, and its performance indexes are shown in Table 4. (4) Tap water. (5) Waste ceramic particles particles: Waste ceramic particles particles produced by Shandong Meitong Road Construction New Materials Co., Ltd. were used. (6) Admixture: Air-entraining high-efficiency water reducer was used (Table 5).

**Table 1:** Chemical composition of cement(%).

Raw material	SiO_2_	Fe_2_O_3_	Al_2_O_3_	CaO	MgO	SO_3_	C_3_A	Loss on ignition
Cement	25.32	5.40	7.25	51.32	3.28	2.09	2.56	1.48

**Table 2:** performance indexes of recycled coarse aggregate.

Void ratio (%)	Moisture content (%)	Water absorption rate (%)	Apparent density (kg/m^3^)	Bulk density (kg/m^3^)
48.31	0.52	5.72	2468	1428

**Table 3:** performance indexes of recycled fine aggregate.

Void ratio (%)	Moisture content (%)	Water absorption rate (%)	Apparent density (kg/m^3^)	Bulk density (kg/m^3^)
40.78	0.71	6.59	2257	1507

**Table 4:** performance indexes of natural coarse aggregate.

Void ratio (%)	Moisture content (%)	Water absorption rate (%)	Apparent density (kg/m^3^)	Bulk density (kg/m^3^)
45.31	0.3	1. 03	2010	1629

**Table 5:** performance indexes of Waste ceramic particless.

Void ratio (%)	Moisture content (%)	Water absorption rate (%)	Apparent density (kg/m^3^)	Bulk density (kg/m^3^)
30.31	0.41	1. 48	2680	1610

### Test scheme design

**Fig. 1:**
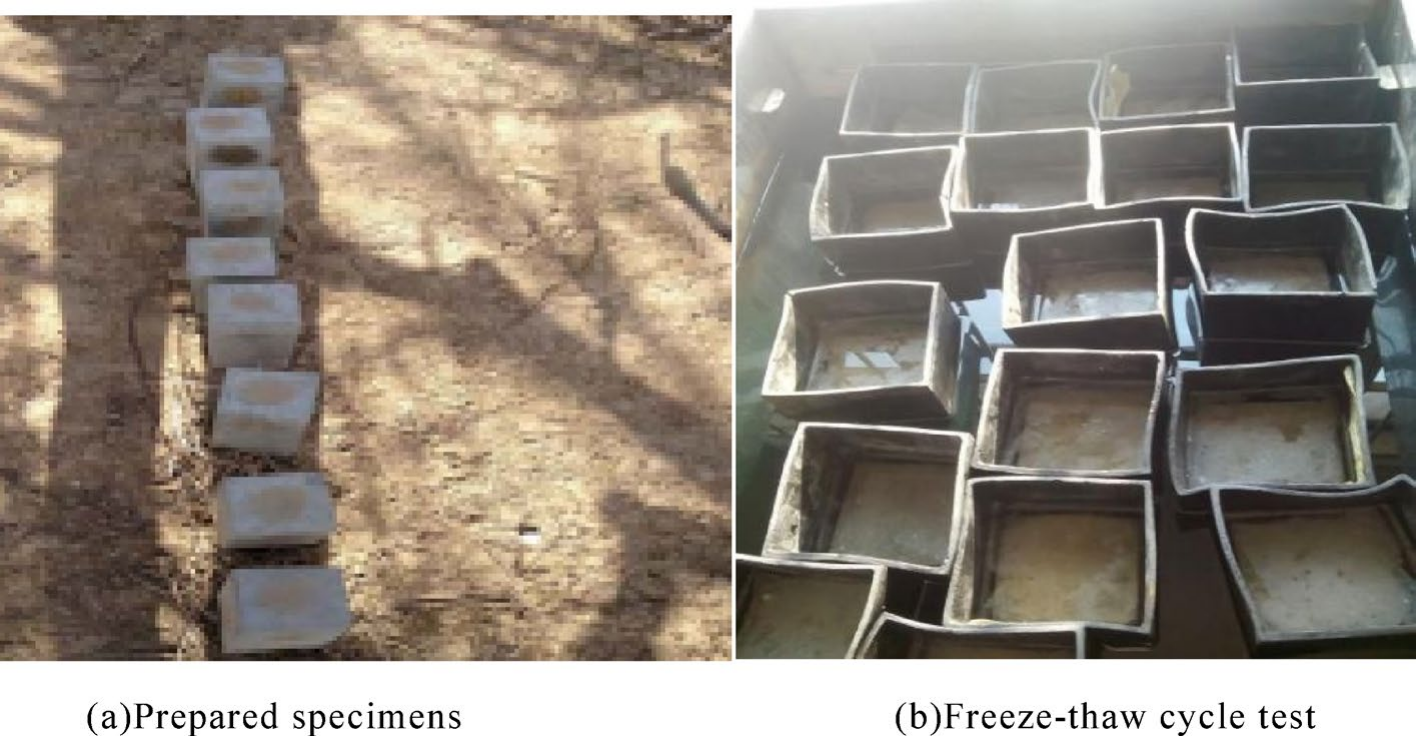
Prepared specimens and freeze–thaw cycle test.

To ensure the scientificity and standardization of the test, the rapid freezing method (operating under water-saturated freezing and water-thawing cyclic conditions) was employed in this study to evaluate the frost resistance of recycled concrete. Specifically, the frost resistance level of the concrete specimens was characterized by the cumulative number of rapid freeze–thaw cycles they could withstand before reaching the failure criteria. All test procedures and specimen specifications strictly complied with the technical requirements specified in the national standard Test Methods for Long-term Performance and Durability of Ordinary Concrete (GB/T 50,082–2009). The central temperature of the specimens was controlled from − 18 (freezing end) to 5 ℃ (thawing end), in full accordance with the standard range of − 18 ℃ ± 2 ℃ to 5 ℃ ± 2 ℃. The heating and cooling rate was about 5.75 ℃ per hour, with one freeze‑thaw cycle finished in 4 h across a 23 ℃ temperature span, which meets the standard limit of 10 ℃/h. A 1‑hour constant temperature duration was applied at both − 18 ℃ and 5 ℃ to ensure uniform temperature distribution inside the specimens as required by the standard. The specimens were fabricated with a standard dimension of 100 mm × 100 mm × 100 mm to meet the requirements of freeze–thaw cycle testing. A total of six mix proportion groups were designed for the test, with three parallel specimens prepared for each group to ensure the reliability and reproducibility of the test results. The specimens underwent standard curing for 24 days prior to the freeze–thaw test. At this stage, the specimens designated for freeze–thaw testing were pre-removed from the standard curing chamber and subsequently subjected to water immersion treatment. During immersion, the water level was maintained 20–30 mm above the top surface of the specimens to ensure complete water saturation of the specimens. The water immersion duration was set at 4 days, and the freeze–thaw cycle test was initiated precisely when the specimens reached the 28-day standard curing age (a critical time point for evaluating concrete mechanical and durability properties).A schematic diagram of the freeze–thaw cycle test setup is presented in Fig. 1, while the detailed mix proportion parameters of each group of Waste ceramic particles aggregate recycled concrete are summarized in Table 6.

**Table 6:** scale of recycled ceramics concrete.

Group	Cement	Natural coarse aggregate	Recycled coarse aggregate	Recycled fine aggregate	Ceramic particles	Water	Superplasticizer (%)	Ceramic particle substitution rate (%)
CZ-0	1	1.61	1.61	1.96	0.00	0.5	1	0
CZ-20	1	1.61	1.61	1.568	0.392	0.5	1	20
CZ-40	1	1.61	1.61	1.176	0.784	0.5	1	40
CZ-60	1	1.61	1.61	0.784	1.176	0.5	1	60
CZ-80	1	1.61	1.61	0.392	1.568	0.5	1	80
CZ-100	1	1.61	1.61	0.00	1.96	0.5	1	100%

Throughout the experimental process, the core temperatures of the specimens were precisely controlled within the preset minimum and maximum limits, with each individual freeze–thaw cycle configured to last 4 h. Performance measurements targeting dynamic elastic modulus and specimen mass were conducted at an interval of every 30 freeze–thaw cycles. Prior to each measurement session, surface contaminants and scum were thoroughly removed, residual surface moisture was wiped off completely, and a comprehensive visual inspection of the specimens’ external appearance was carried out. Subsequent to these pretreatment steps, calculations of the relative dynamic elastic modulus and mass loss rate were performed, with both parameters being designated as the core evaluation metrics for assessing the freeze–thaw resistance of the concrete. The experiment was terminated immediately once any of the following termination criteria were met: the cumulative number of freeze–thaw cycles reached 300 times; the relative dynamic elastic modulus declined to 60% of its initial value; or the mass loss rate exceeded the threshold of 5%.

Based on the collected experimental data, the mix proportion with optimal freeze–thaw durability was determined. The Wiener mathematical probability distribution model was applied to analyze the correlation between cycle count and specimen reliability, thereby forecasting the service life of specimens with this optimal mix proportion under freeze–thaw conditions.

## Test results and analysis

### Mode of failure

**Fig. 2:**
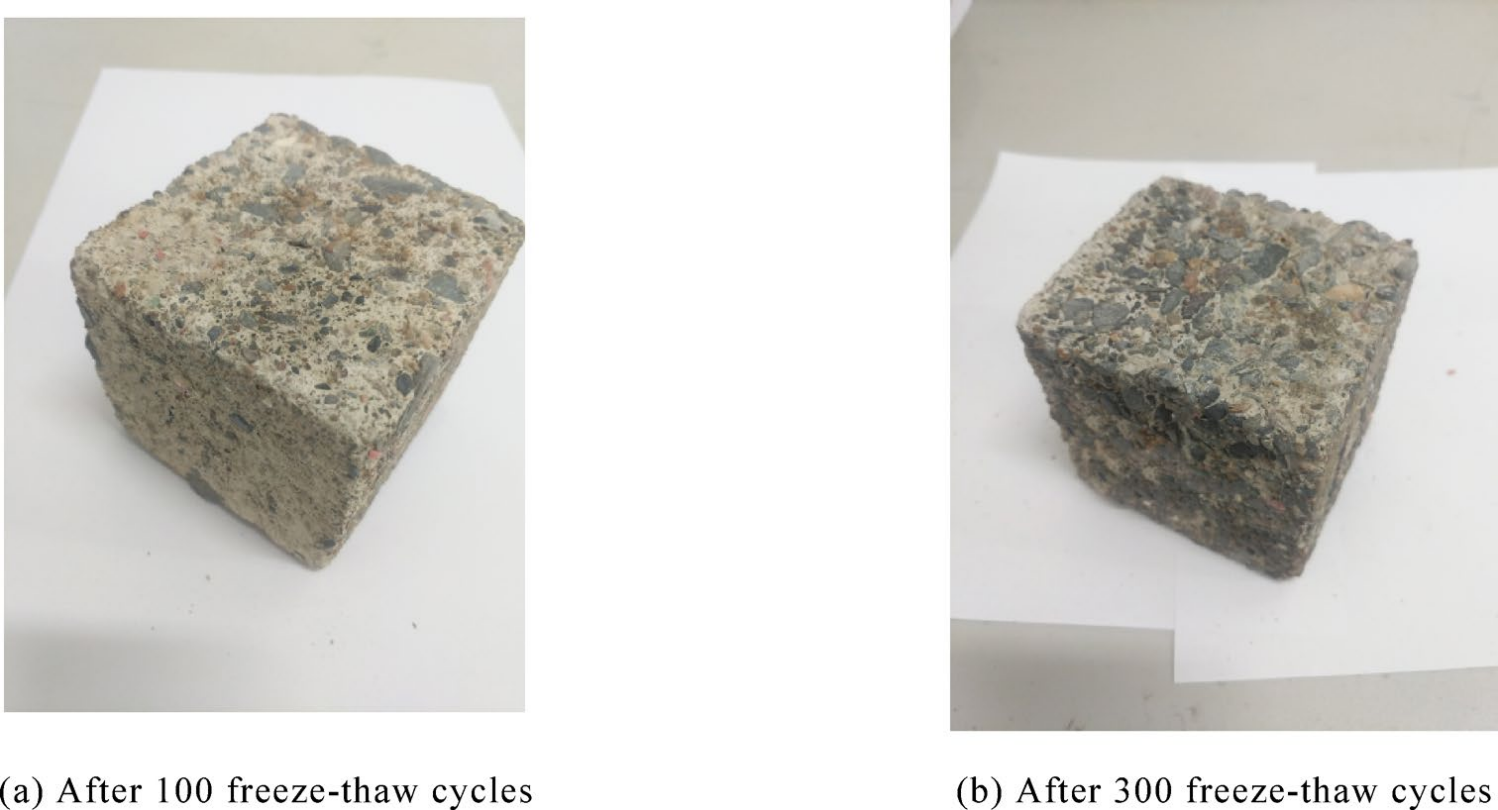
Macroscopic failure mode.

Figure 2a presents the specimen’s morphology after 100 freeze–thaw cycles, while Fig. 2b illustrates its state following 300 cycles. After 100 cycles, the specimen retains its overall geometric integrity, with only slight spalling of surface cement mortar at edges and corners—exposing small amounts of fine aggregates and ceramic particles locally. Its surface features a light gray base with distinct mottled textures of ceramic aggregates, and no macroscopic cracks or obvious structural defects are detected. This observation indicates that damage under 100 cycles is primarily confined to the deterioration of the surface interfacial transition zone (ITZ), without causing significant harm to the internal structure. In contrast, the specimen’s appearance after 300 rapid freeze–thaw cycles shows drastically worsened damage: edges and corners exhibit obvious chipping and defects, large areas of surface cement mortar have peeled off, and numerous internal ceramic and coarse aggregates are exposed with accompanying particle shedding. The specimen’s surface has darkened to dark grayish brown, with reticulated microcracks initiating and spreading in local regions, and its overall geometric integrity has been compromised. This phenomenon reveals that as freeze–thaw cycles accumulate, internal microcracks continue to expand, interfacial bonding gradually deteriorates, and damage penetrates from the surface to the internal structure—pushing the specimen into a phase of accelerated damage progression.

**Fig. 3:**
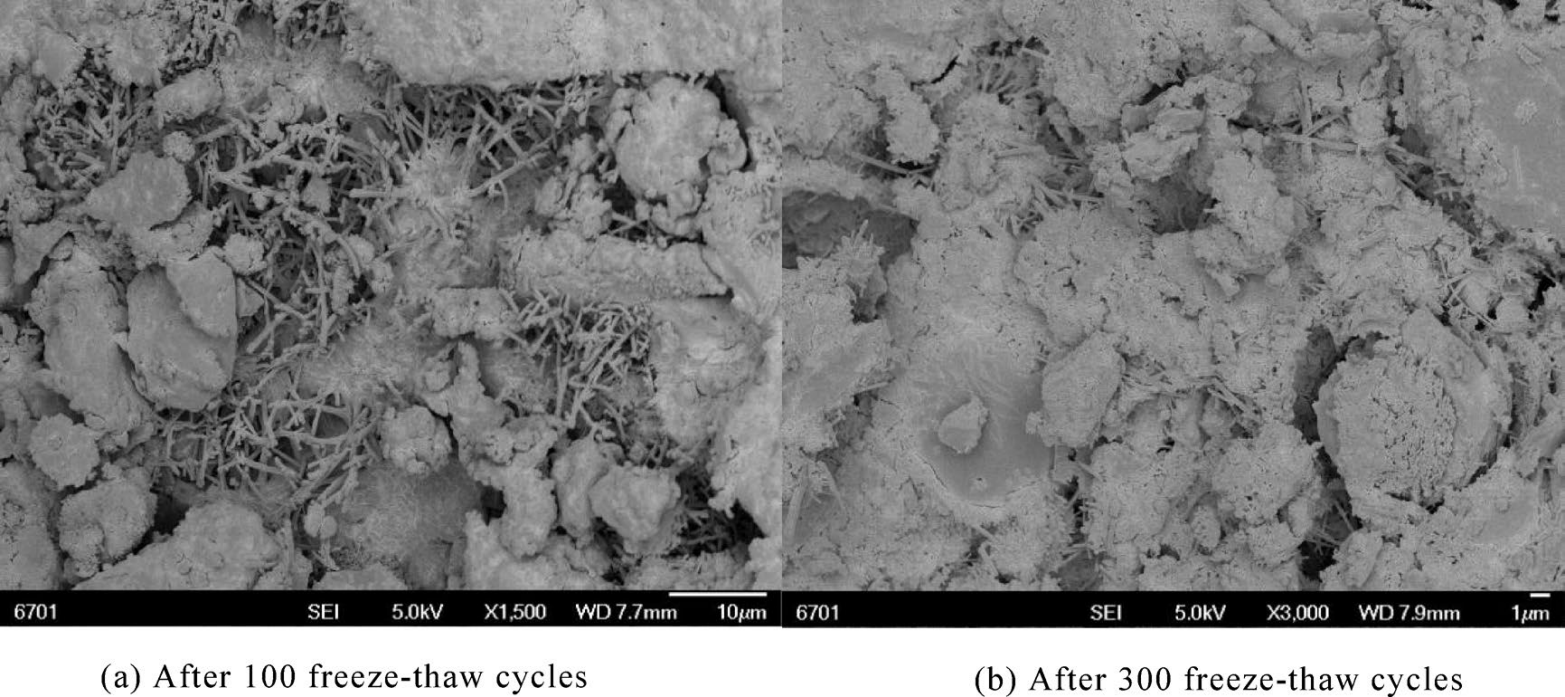
Microscopic failure mode.

The scanning electron microscopy (SEM) micrographs of Fig. 3a (after 100 freeze–thaw cycles) and Fig. 3b (after 300 freeze–thaw cycles) visually characterize the microstructural evolution of concrete under varying freeze–thaw exposure:

Following 100 freeze–thaw cycles, the pore size within the concrete matrix remains relatively small and uniformly distributed. At the matrix-aggregate interface and between pores, an extensive network of acicular, rod-like, and fibrous hydration products (e.g., ettringite, calcium silicate hydrate gel) is observed, forming a continuous cementitious framework. This microstructural feature indicates that freeze–thaw action at this stage induces only minor degradation of the surface interfacial transition zone (ITZ), while internal hydration products retain sufficient micro-bonding strength to maintain matrix integrity. No significant structural dissociation is evident within the pores, which aligns with the macroscopic damage state of slight surface mortar spalling and absence of macroscopic cracks.

After 300 freeze–thaw cycles, the concrete microstructure exhibits substantial deterioration: pore dimensions increase markedly and form interconnected networks, the original hydration product cementitious framework is fully disintegrated, and continuous hydration product bonding (both between pores and within the matrix) is absent—leaving only loose granular matrix debris. This phenomenon arises from the cumulative “freeze-swelling–thaw-shrinking” effect of pore water, which drives progressive microcrack propagation; concurrently, hydration products undergo physical spalling and chemical decomposition under freeze–thaw stress, resulting in the loss of interfacial bonding and matrix strength. Macroscopically, this manifests as an accelerated damage phase characterized by “exposed aggregates and compromised structural integrity”.

With increasing freeze–thaw cycles, the cascading process (pore water freeze-swelling stress → microcrack propagation → hydration product disintegration → pore connectivity) transforms the concrete microstructure from a “densely cemented” state to one of “loose dissociation”. This directly correlates with the macroscopic performance degradation (e.g., reduced relative dynamic elastic modulus, elevated mass loss) and validates the freeze–thaw damage mechanism: a progression from “surface ITZ degradation” to “permeation into the internal structure”.

In addition, the interfacial defects at high ceramic contents (> 40%) by combining microscopic tests and macroscopic performance; specifically, the thickness of the interfacial transition zone (ITZ) was statistically measured by SEM, showing that the ITZ thickness of the 20% replacement group was about 20–30 μm, while that of the 80% replacement group increased to 50–70 μm with a porosity increase of approximately 40%. According to SEM image analysis using Image-Pro Plus software, the microcrack density of the 100% replacement group reached 8–10 cracks/mm^2^ after 100 cycles, about four times that of the 20% replacement group (2–3 cracks/mm^2^). The interfacial bond strength was indirectly characterized by the attenuation rate of the relative dynamic elastic modulus, and the modulus attenuation rate of the high ceramic content groups (> 40%) was 2.5–3 times that of the 20% replacement group, indicating that interfacial bond failure was the main cause of performance degradation.

### Damage analysis based on dynamic elastic modulus

**Fig. 4:**
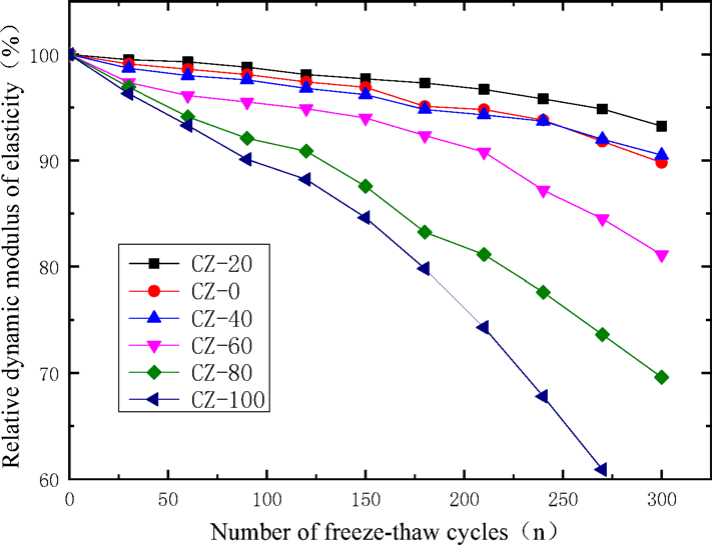
Variation curves of dynamic elastic modulus with freeze–thaw cycles.

The relative dynamic elastic modulus is determined by the change of transverse fundamental frequency, reflecting the degree of concrete damage after freeze–thaw cycles. It is calculated by formula (1) :

1 $$P_{ni} = \frac{{E_{ni} }}{{E_{n0} }} \times {1}00\%$$

where: $E_{n0}$,$E_{ni}$,$P_{ni}$ denote the initial dynamic elastic modulus, the dynamic elastic modulus of the *n*-th specimen after the *i*-th freeze–thaw cycle, and the corresponding relative dynamic elastic modulus of the same specimen following the *i*-th freeze–thaw cycle, respectively. The variation curves illustrating the correlation between the relative dynamic elastic modulus of Waste ceramic particles aggregate recycled concrete and the cumulative number of freeze–thaw cycles are presented in Fig. 4.

Figure 4 reveals that: (1) The dynamic elastic modulus of all specimens decreases with the increase of the number of freeze–thaw cycles, that is, under the condition of freeze–thaw cycles, the damage degree of the specimens gradually increases. (2) At a Waste ceramic particles particle incorporation level of 20%, the corresponding recycled concrete (designated as CZ-20) exhibits the optimal freeze–thaw resistance among all tested groups. This superior performance can be attributed to the intrinsic material properties of Waste ceramic particles particles: compared with conventional recycled fine aggregates, these particles possess lower moisture absorption capacity and porosity. Such characteristics effectively reduce the overall moisture content and internal porosity of the prepared concrete specimens, thereby minimizing the volume expansion induced by water freezing within the material pores during freeze–thaw cycles. This mitigation of internal structural damage directly contributes to the enhanced freeze–thaw durability of the CZ-20 concrete^19^. (3) With the increase of Waste ceramic particles particles, when the content exceeds 40%, the degree of freeze–thaw damage increases significantly. The reason is that the surface of Waste ceramic particles particles contains glaze. Although their moisture content and porosity are lower than those of recycled fine aggregates, their bonding degree with concrete is poor. Excessive Waste ceramic particles particles will lead to excessive voids during the bonding process of recycled concrete. Under freeze–thaw conditions, the water in these pores condenses into solid ice, and the volume increase produces expansion stress, which reduces the frost resistance. When the specimens are subjected to repeated freeze–thaw cycles, a vicious circle is formed due to the above reasons, resulting in damage phenomena such as peeling and spalling on the surface of the specimens and accelerating the damage of the specimens. When the content of Waste ceramic particless is 80%, the freeze–thaw damage rate is significantly accelerated, the damage is serious, and the specimens cannot meet the frost resistance requirements of building materials after 300 freeze–thaw cycles; when the content reaches 100%, the specimens can only withstand 200 freeze–thaw cycles or even fewer.

### Damage analysis based on mass loss

**Fig. 5:**
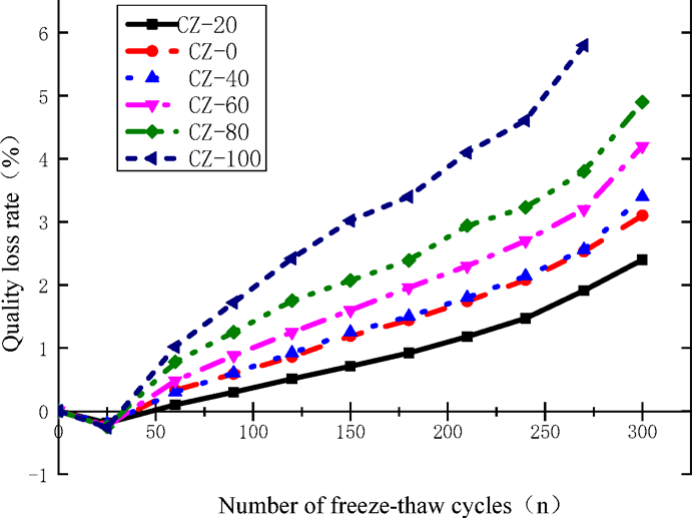
Variation rule of mass loss rate with freeze–thaw cycles.

The mass loss rate of cube specimens of Waste ceramic particles aggregate recycled concrete after every 30 freeze–thaw cycles is calculated by formula (2):

2 $$W_{ni} = \frac{{M_{n0} - M_{ni} }}{{M_{n0} }}$$

where:*M*_n0_,*M*_ni_,*W*_ni_ denote the initial mass of the n-th specimen, its mass following the i-th freeze–thaw cycle, and the corresponding mass loss rate of this specimen after the i-th freeze–thaw cycle, respectively. The variation curves depicting the relationship between the mass of Waste ceramic particles aggregate recycled concrete specimens across all test groups and the cumulative number of freeze–thaw cycles are presented in Fig. 5.

Figure 5 shows that: (1) After 300 freeze–thaw cycles, the specimen with 20% Waste ceramic particles content (CZ-20) has the smallest mass loss rate, and the mass loss rate of CZ-40 is the closest to that of CZ-0. The mass loss rates of specimens with different substitution rates in ascending order are CZ-20, CZ-0, CZ-40, CZ-60, CZ-80, and CZ-100. This is consistent with the change of relative dynamic elastic modulus. (2) Approximately around 25 freeze–thaw cycles, the mass loss rate is negative, that is, the mass of each group of specimens increases. The main reasons are: before the test, the specimens need to be completely immersed in water for 4 days. During this process, external water enters the interior of concrete through capillary channels formed by initial defects such as microcracks until saturation. Subsequently, the freeze–thaw test starts, and the specimens are in the cycle of water freezing and thawing with temperature changes in the saturated state. The mass change is mainly due to two reasons: one is the erosion of surface particles, and the other is the continuous intrusion of water. With the increase of the number of freeze–thaw cycles, the free water retained in the internal voids of concrete freezes at low temperatures, and the capillary pore walls are subjected to the superposition of frost heave pressure and osmotic pressure, resulting in the continuous accumulation and expansion of microcracks, so that external water continues to intrude into the interior of the specimens. At the same time, a small amount of fine cement paste particles fall off the surface of the specimens, and a small number of tiny pores appear. Approximately at 25 cycles, due to the small number of cycles, the amount and weight of surface detached particles are small, which is less than the mass of water intruding into the interior of the specimens, so the mass loss rate is negative. After that, with the increase of the number of freeze–thaw cycles, the degree of surface erosion of the specimens intensifies, the weight of detached particles gradually exceeds the weight of intruding water, and the mass loss rate begins to increase gradually. With the spalling of aggregates, the mass loss rate becomes larger and larger^20^.

In conclusion, under cyclic freeze–thaw exposure conditions, the recycled concrete incorporating Waste ceramic particles aggregates achieves optimal freeze–thaw resistance when the incorporation level of Waste ceramic particles particles is set at 20%. Beyond this optimal dosage threshold, a pronounced negative correlation emerges between the content of Waste ceramic particles particles and the freeze–thaw durability of the resultant recycled concrete: the material’s resistance to freeze–thaw damage declines sharply with the continuous increase in ceramic tile particle content. Notably, when Waste ceramic particles particles completely replace the conventional recycled fine aggregates (i.e., at a 100% incorporation level), the corresponding concrete specimens suffer irreversible structural damage well before the freeze–thaw cycle count reaches 300, indicating a dramatic deterioration in their frost resistance performance.

## Reliability analysis of freeze–thaw cycles

Reliability analysis constitutes a critical technical approach in engineering evaluation, which focuses on quantifying and assessing the reliability level of products or structural components within a specified service period by systematically analyzing their performance degradation data under specific working conditions. In this study, the Wiener distribution probability model—widely recognized for its superior adaptability in describing gradual degradation processes—is adopted for data processing and analytical modeling.

Taking the mass loss rate and dynamic elastic modulus, which are highly sensitive to freeze–thaw cycle effects, as the core performance evaluation indicators, this research constructs a comprehensive performance degradation model that characterizes the evolutionary law of specimen performance with the accumulation of freeze–thaw cycles.This function not only effectively reflects the remaining service life of the specimens under freeze–thaw environmental conditions but also enables a comparative analysis of the sensitivity differences between the two evaluation indicators in responding to freeze–thaw cycle-induced damage.

It is anticipated that the analytical framework and technical method proposed in this paper can be extended to practical engineering scenarios, providing a scientific and reliable technical basis for the testing, evaluation, and service life prediction of similar materials or structural components in freeze–thaw-prone environments^21^. From the theoretical perspective of reliability modeling, when the performance degradation process of a product conforms to the univariate Wiener process, the service life T of the product can be defined as the time point at which the cumulative amount of performance degradation first reaches the predefined failure threshold, specifically expressed as:

3 $$T = inf\{ t|X(t) = D_{\begin{subarray}{l} f \\ \end{subarray} } ,t \ge 0\}$$

where: *D*_f_ is the failure threshold of specimen degradation. In accordance with the technical specifications outlined in the national standard"Test Methods for Long-term Performance and Durability of Ordinary Concrete" (GB/T 50,082–2009), a concrete specimen is deemed to have reached the failure criterion when either its mass loss rate exceeds 5% or its relative dynamic elastic modulus experiences a reduction of 40%^18^. Consequently, in the context of reliability analysis, the critical failure threshold *D*_f_ is assigned a value of 0.4 when calculating the time to failure based on the degradation of dynamic elastic modulus. Conversely, when evaluating the time to failure with respect to mass loss, the failure threshold *D*_f_ is set at 0.05 to align with the aforementioned standard requirements.

For a certain sample *i* among *n* samples, at the initial moment *t*_0_, the performance degradation of the product is assumed to be zero, i.e., *X*_i0_ = 0. The performance degradation of the product is then measured at a series of discrete time points *t*_*1*_*,……, t*_*mi*_, and the corresponding measured values are denoted as $X_{i1} ,...,X_{{im_{j} }}$. Let $\Delta x_{ij} = X_{ij} - X_{i(j - 1)}$ represent the increment of performance degradation for the i-th sample between the two adjacent measurement times*t*_*j-1*_ and *t*_*j*_, i.e., $\Delta x_{ij} = X_{ij} - X_{i(j - 1)}$.According to the basic properties of the Wiener process, the following relationships hold:

$$\Delta x_{ij} \sim N(u\Delta t_{j} ,\sigma^{2} \Delta t_{j} )$$

where, $$\Delta t_{j} = t_{j} - t_{(j - 1)} ;i = 1,...,n;j = 1,...,m_{j}$$.

The likelihood function obtained from the performance degradation data is:

4 $$L(\mu ,\sigma^{{2}} ) = \prod {_{i - 1}^{n} } \prod {_{j = 1}^{{m_{i} }} } \frac{{1}}{{\sqrt {{2}\sigma^{{2}} \pi \Delta {\mathrm{t}}_{j} } }}{\mathrm{exp}}[ - \frac{{(\Delta x_{ij} - u\Delta t_{j} )^{2} }}{{{2}\sigma^{{2}} \Delta {\mathrm{t}}_{j} }}]$$

The maximum likelihood estimates of parameters $u$ and $\sigma^{{2}}$ can be directly obtained from formula (4) as:

5 $$\mathop u\limits^{ \wedge } = \frac{{\sum {_{i = 1}^{n} x_{{im_{i} }} } }}{{\sum {_{i = 1}^{n} t_{{m_{i} }} } }},\mathop {\mathop {\sigma^{2} }\limits^{ \wedge } = }\limits^{{}} \frac{1}{{\sum {_{i = 1}^{n} m_{i} } }}[\sum {_{i = 1}^{n} \sum {_{j = 1}^{{m_{i} }} \frac{{(\Delta x_{ij} )^{2} }}{{\Delta t_{j} }} - \frac{{(\sum {_{i = 1}^{n} x_{{im_{i} }} )^{2} } }}{{\sum {_{i = 1}^{n} t_{{m_{i} }} } }}} } ]$$

The point estimate of reliability at time *t* can be obtained as:

6 $$R(t) = 1 - F(t) = \Phi \left( {\frac{{D_{f} - \mathop {\mu t}\limits^{ \wedge } }}{{\mathop \sigma \limits^{ \wedge } \sqrt t }}} \right) - \exp \left( {\frac{{2\mathop \mu \limits^{ \wedge } D_{f} }}{{\mathop \sigma \limits^{ \wedge 2} }}} \right)\Phi \left( {\frac{{ - D_{f} - \mathop {\mu t}\limits^{ \wedge } }}{{\mathop \sigma \limits^{ \wedge } \sqrt t }}} \right)$$

Based on the optimal mix ratio for the freeze–thaw cycle resistance of Waste ceramic particles particle recycled concrete, that is, the substitution rate of Waste ceramic particles particles is 20%, the Wiener reliability analysis is carried out with this mix ratio. The data obtained after every 30 freeze–thaw cycles are counted, and the time-dependent degradation data of durability performance are shown in Table 7:

**Table 7:** Degradation of durability performance.

Number of cycles		30	60		90		120	150	180	210	240	270	300
Relative dynamic elastic modulus loss D(n_ij_)	D(n_1j_)	0.0139	0.0269		0.0351		0.0485	0.0628	0.0770	0.0878	0.1028	0.1368	0.1889
	D(n_2j_)	0.0170	0.0312		0.0412		0.0406	0.0610	0.0712	0.0931	0.1410	0.1435	0.2045
	D(n_3j_)	0.0110	0.0216		0.0302		0.0401	0.0696	0.0815	0.0813	0.1241	0.1315	0.1962
Quality loss W(n_ij_)	W(n_1j_)	− 0.0012	0.0025		0.0043		0.0070	0.0100	0.0151	0.0225	0.0338	0.0443	0.0550
	W(n_2j_)	− 0.0001	0.0019		0.0030		0.0067	0.0123	0.0168	0.0209	0.0351	0.0461	0.0515
	W(n_3j_)	− 0.0015	0.0014		0.0052		0.0060	0.0112	0.0141	0.0241	0.0373	0.0399	0.0560

Based on the aforementioned research conclusions and analytical results, it can be inferred that the variations in dynamic elastic modulus and mass loss of specimens during the freeze-thaw cycle test essentially reflect the cumulative damage evolution process of the material internal structure. Specifically, as the freeze-thaw cycles accumulate, the internal microcracks of the specimens continue to initiate, expand, and connect, which directly leads to the degradation of dynamic elastic modulus and the loss of mass. When the degree of such performance degradation exceeds the threshold that the material can withstand, the structural integrity of the specimens will be damaged, ultimately resulting in failure.

To accurately characterize this damage evolution law, this study takes the absolute value of the performance degradation amount Δ*x* between adjacent measurement intervals as the core characterization parameter for constructing the performance degradation model. This parameter can effectively capture the incremental damage degree of the specimens in each test stage, thereby realizing the refined description of the continuous degradation process of the material under freeze-thaw action. Building upon this analytical framework, a reliability function is further formulated to quantitatively characterize the intrinsic correlation between the reliability level of the test specimens and the cumulative number of freeze-thaw cycles. This function not only enables the precise prediction of the material’s remaining service life under cyclic freeze-thaw exposure but also provides a direct theoretical basis for the durability evaluation and service life prediction of such materials in practical engineering environments subjected to freeze-thaw cycles.^22^. The specific values of the performance degradation amount Δx between adjacent measurement times are detailed in Table 8.

**Table 8:** performance degradation at adjacent measurement times $\Delta x$.

Number of cycles		30	60	90	120	150	180	210	240	270	300
Relative dynamic elastic modulus loss _D(nij)_	ΔD(n1j)	0.02	0.03	0.06	0.04	0.10	0.19	0.02	0.07	0.1	0.22
	ΔD(n2j)	0.02	0.02	0.03	0.11	0.05	0.18	0.07	0.05	0.11	0.15
	ΔD(n3j)	0.01	0.04	0.07	0.02	0.08	0.14	0.02	0.05	0.09	0.26
Quality loss W(n_ij_)	$\Delta$ W(n_1j_)	0.001	0.003	0.008	0.009	0.012	0.014	0.021	0.009	0.015	0.032
	$\Delta$ W(n_2j_)	0.001	0.002	0.010	0.012	0.009	0.014	0.019	0.007	0.019	0.028
	$\Delta$ W(n_3j_)	0.002	0.004	0.006	0.011	0.016	0.012	0.017	0.010	0.016	0.034

**Fig. 6:**
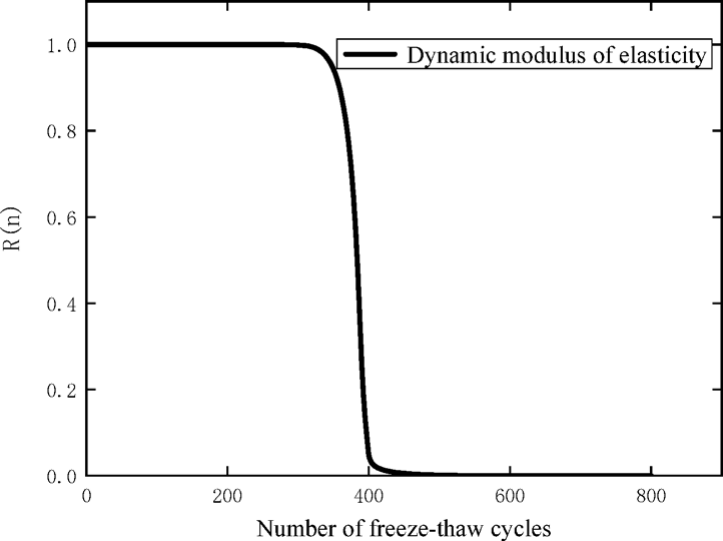
Reliability curve of specimen with freeze–thaw cycles.

Based on the relative dynamic elastic modulus loss values at adjacent times in Table 8, the maximum likelihood estimates of parameters *m* and *s*^2^ can be calculated by formulas (4) and (5) as $\mathop \mu \limits^{ \wedge } = {0}{\mathrm{.098,}}\mathop {\sigma^{{2}} }\limits^{ \wedge } = {0}{\mathrm{.0010298886}}$ . Substituting them into formula (6), the reliability curve of the specimens with the number of freeze–thaw cycles can be obtained as shown in Fig. 6.

As can be observed from Fig. 6, the reliability evolution law of the CZ-20 specimen during the freeze–thaw cycle test presents a distinct phased characteristic. In the initial stage of the test, the reliability of the specimen maintains a nearly stable state with the increase of freeze–thaw cycle times, and no obvious downward trend can be detected before the cycle number reaches approximately 285. However, once the freeze–thaw cycles exceed the threshold of 285 times, the reliability of the specimen declines sharply as the test progresses, indicating that the internal damage of the material enters an accelerated development stage. When the reliability of the specimen drops to 0, which means the material suffers complete functional failure, the corresponding maximum number of freeze–thaw cycles that the specimen can withstand is 415. For the sake of engineering safety application, the reliability threshold of 0.6 is defined as the critical value for evaluating the service performance of the specimen; under this standard, the CZ-20 specimen can resist 383 freeze–thaw cycles.

In this research section, the mass loss of the specimen is selected as the core index to characterize the performance degradation degree, and a targeted reliability analysis is carried out for the CZ-20 specimen under freeze–thaw cycle conditions.

**Fig. 7:**
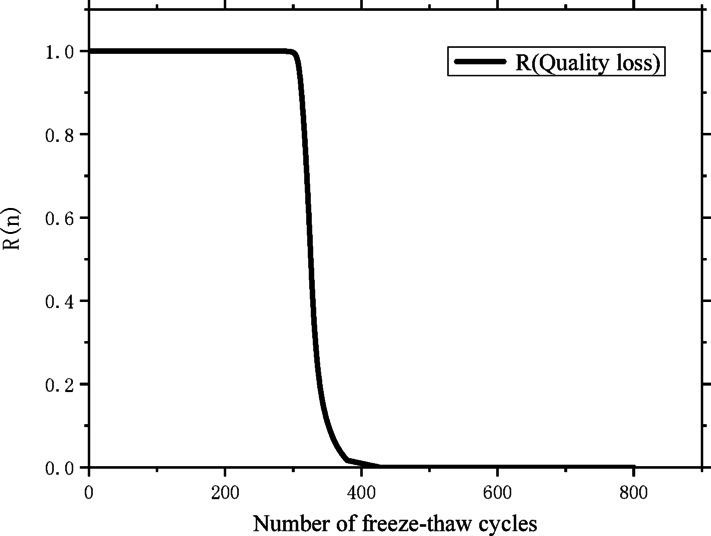
Reliability curve of specimen with freeze–thaw cycles.

In a similar analytical approach, based on the mass loss data of the specimen between adjacent measurement time nodes presented in Table 8, the maximum likelihood estimation method is adopted to calculate the values of parameters *m* and *s*^2^ by applying formula (4) and formula (5) respectively ($\mathop \mu \limits^{ \wedge } = {0}{\mathrm{.095,}}\mathop {\sigma^{{2}} }\limits^{ \wedge } = {0}{\mathrm{.00105092}}$). After obtaining the accurate estimated values of the two key parameters, they are substituted into formula (6) for further derivation and calculation. On this basis, the reliability curve reflecting the correlation between the reliability of the CZ-20 specimen and the number of freeze–thaw cycles is finally constructed, and the specific change trend of the curve is displayed in Fig. 7.

It can be further derived from the reliability analysis results that when the reliability index of the specimen drops to 0, corresponding to the state of complete functional failure of the material, the maximum number of freeze–thaw cycles that the specimen can withstand is approximately 398. From the perspective of practical engineering safety application, taking the reliability index of 0.6 as the critical threshold for evaluating the service performance of the specimen, the number of freeze–thaw cycles that the specimen can resist under this safety standard is 334.

**Fig. 8:**
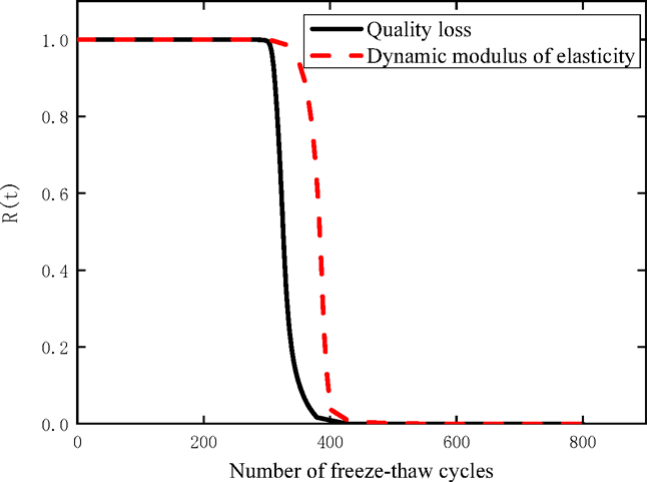
Reliability curve merging diagram of specimen with freeze–thaw cycles.

It is worth noting that there are significant differences in the outcomes of the freeze–thaw cycle reliability analysis when using the two evaluation indexes, namely dynamic elastic modulus and mass loss rate. This discrepancy is mainly due to the fact that the two indexes respond to the internal damage evolution of the material with different sensitivity and characterization focus during the freeze–thaw cycle process. To comprehensively and systematically characterize the reliability evolution law of the specimen under freeze–thaw action, this study conducts an integrated analysis of the reliability analysis results obtained from the two indexes, and further merges the corresponding reliability curves to form a comprehensive and unified reliability evaluation curve, as shown in Fig. 8.

This test demonstrates that under freeze–thaw cycling conditions, the Wiener distribution exhibits excellent applicability in characterizing the degradation process of freeze–thaw resistance of the CZ-20 specimen. Among the durability evaluation indicators of recycled concrete in freeze–thaw environments, the mass loss rate shows higher sensitivity to freeze–thaw-induced damage compared to the dynamic elastic modulus. Additionally, the two indicators follow a competing failure mechanism—specifically, the specimen is deemed to have failed once either of the two indicators reaches the predefined failure threshold. Based on the analysis results, the earliest failure of the CZ-20 specimen occurs after undergoing 398 freeze–thaw cycles.

## Comparison with previous studies

The study results show that replacing 20% of recycled fine aggregate with ceramic particles yields the best freeze–thaw resistance, which matches what Siddique et al.^9^ found—proper amounts of ceramic waste do improve concrete durability. Ceramic particles have low water absorption (1.48%) and porosity (30.31%) (Table 5), so they fill gaps in the concrete matrix and reduce damage from freeze–thaw cycles. This is also consistent with Dobiszewska et al.^10^, who noted that ceramic aggregates help refine the internal pore structure of concrete. Saber et al.^11^ reported an optimal replacement rate of 25% when using ceramic waste to replace natural coarse aggregate, which is a bit higher than our 20%. This difference mainly comes from the type of aggregate being replaced (natural coarse vs. recycled fine) and slight variations in mix design, such as the water-cement ratio. In terms of how damage develops, we found that freeze–thaw harm starts at the surface interface transition zone (ITZ), which is similar to Otsuki et al.’s^7^ findings on recycled aggregate concrete. What’s different is that our samples with 20% ceramic particles had better early resistance—after 100 freeze–thaw cycles, their relative dynamic elastic modulus stayed above 90%.

## Conclusions

1. Comprehensive analysis of the test data for all specimen groups reveals a distinct dose-dependent relationship between Waste ceramic particles particle content and the freeze–thaw resistance of recycled concrete. Specifically, the recycled concrete achieves the maximum freeze–thaw resistance when the Waste ceramic particles particle dosage is set at 20%. At a 40% content, the freeze–thaw resistance of the composite concrete is comparable to that of conventional recycled concrete without ceramic tile aggregates. A critical inflection point appears when the content exceeds 40%: the freeze–thaw resistance of the recycled concrete declines sharply with the further increase in ceramic tile particle dosage. Notably, when Waste ceramic particless fully replace recycled fine aggregates (i.e., the replacement ratio reaches 100%), the resultant recycled concrete fails to sustain even 300 freeze–thaw cycles, signifying a drastic deterioration in its frost durability.
2. Dynamic performance monitoring of all specimens during freeze–thaw cycling demonstrates divergent evolutionary trends for different durability indicators. The relative dynamic elastic modulus of all specimens shows a monotonous decreasing trend with the accumulation of freeze–thaw cycles, which is essentially induced by the initiation, propagation and coalescence of internal microcracks under cyclic freeze–thaw stress. In contrast, the mass of the specimens exhibits a biphasic variation pattern—increasing slightly in the early stage and then decreasing continuously as the test proceeds. The initial mass gain is primarily attributed to the water absorption of microcracks formed in the early freeze–thaw stage, while the subsequent mass loss results from the spalling of surface mortar and aggregate particles due to aggravated internal damage. The mass of the specimens reaches its peak value when the freeze–thaw cycle number approaches 25.
3. The Wiener distribution model exhibits high predictive accuracy and robustness in forecasting the service life of ceramic tile particle-reinforced recycled concrete under freeze–thaw conditions. Based on the model simulation results, for the optimal mix proportion with 20% Waste ceramic particles particles, the reliability of the recycled concrete remains stable in the early freeze–thaw stage and does not undergo a sharp decline until the cycle number reaches at least 265. When the reliability index drops to 0 (defined as the complete functional failure of the specimen), the minimum freeze–thaw cycle number that the specimen can withstand is 398. From the perspective of engineering safety design, taking a reliability index of 0.6 as the serviceability limit state, the minimum freeze–thaw cycle number that the specimen can resist under this safety criterion is 334.
4. This study identifies 20% ceramic particle replacement as optimal for recycled concrete in cold, high-altitude areas, validated via freeze–thaw tests and Wiener model predictions. Limited to single-factor freeze–thaw analysis, future research will integrate carbonation resistance, Rapid Chloride Migration (RCM), and sorptivity tests to quantify long-term transport properties^23^. A multi-factor durability prediction model will be established, with ceramic particle surface modification to improve interfacial transition zone (ITZ) performance^24^. These efforts aim to support large-scale, high-value application of ceramic waste in cold-region infrastructure.

## Data Availability

The datasets used and analysed during the current study available from the corresponding author on reasonable request.
